# Neural Activities Underlying the Feedback Express Salience Prediction Errors for Appetitive and Aversive Stimuli

**DOI:** 10.1038/srep34032

**Published:** 2016-10-03

**Authors:** Yan Gu, Xueping Hu, Weigang Pan, Chun Yang, Lijun Wang, Yiyuan Li, Antao Chen

**Affiliations:** 1 Key Laboratory of Cognition and Personality of Ministry of Education, Faculty of Psychology, Southwest University, Chongqing, China; 2 School of Economics & Management, Southwest Jiaotong University, China

## Abstract

Feedback information is essential for us to adapt appropriately to the environment. The feedback-related negativity (FRN), a frontocentral negative deflection after the delivery of feedback, has been found to be larger for outcomes that are worse than expected, and it reflects a reward prediction error derived from the midbrain dopaminergic projections to the anterior cingulate cortex (ACC), as stated in reinforcement learning theory. In contrast, the prediction of response-outcome (PRO) model claims that the neural activity in the mediofrontal cortex (mPFC), especially the ACC, is sensitive to the violation of expectancy, irrespective of the valence of feedback. Additionally, increasing evidence has demonstrated significant activities in the striatum, anterior insula and occipital lobe for unexpected outcomes independently of their valence. Thus, the neural mechanism of the feedback remains under dispute. Here, we investigated the feedback with monetary reward and electrical pain shock in one task via functional magnetic resonance imaging. The results revealed significant prediction-error-related activities in the bilateral fusiform gyrus, right middle frontal gyrus and left cingulate gyrus for both money and pain. This implies that some regions underlying the feedback may signal a salience prediction error rather than a reward prediction error.

When facing an upcoming event, we usually make a prediction about it and rapidly compare the actual value with predicted value when the feedback is received, producing an error signal (prediction error, PE), which will facilitate a more accurate prediction the next time. There has been a long-term interest in feedback. A prominent early study found a negative deflection in event-related potentials when the feedback implied incorrect performance^1^ (i.e., feedback-related negativity, FRN). This deflection peaks at approximately 250–300 ms after the onset of the feedback and is likely to be derived from the anterior cingulate cortex (ACC) or a more distributed source in supplementary motor areas^2^,^3^. As a signature of the feedback, the FRN is deemed to be sensitive to negative feedback and to reflect the binary (good/bad) evaluation of the outcome^4^. The dominant reinforcement-learning error-related negativity (RL-ERN) theory about the FRN further states that it reflects a reward prediction error (RPE) linked to dopaminergic neurons^5^,^6^. When an appetitive event was unexpectedly delivered or an aversive event was unexpectedly omitted (positive prediction error), the dopaminergic neurons exhibited increased phasic firing, but they showed decreased phasic firing for the unexpected omission of an appetitive event or the unexpected delivery of an aversive event (negative prediction error)^7^,^8^,^9^. Accordingly, the neural mechanism of the feedback is sensitive to the feedback valence, and the PE signal should be opposite for appetitive and aversive stimuli.

Rather than the reward prediction error proposed by the RL-ERN theory, other studies suggest a salience prediction error (SPE), which reflects the unexpectedness of the feedback. Strikingly, the FRN has been elicited by both unexpected positive and unexpected negative feedback^10^,^11^. Talmi *et al.*^12^ found that unexpected reward omission yielded a more negative deflection than unexpected reward delivery. Importantly, they also observed a more negative deflection for unexpected pain omission than unexpected pain delivery, which strongly refuted the RL-ERN theory and suggested that the FRN should signal a SPE. Additionally, some researchers had questioned the RPE hypothesis by pointing out that the phasic responses of dopaminergic neurons were diverse^13^,^14^. Specifically, while some neurons were excited by appetitive stimuli and inhibited by aversive stimuli, others were excited by both.

Furthermore, as stated by the prediction of response-outcome (PRO) theory, the mPFC, particularly the dorsal ACC and the pre-supplementary motor area, may play general roles in detecting discrepancies between the expected and actual outcomes and are sensitive to the unexpectedness of feedback, regardless of its valence^15^,^16^,^17^,^18^. Ferdinand *et al.*^19^ disentangled the effect of valence and expectancy using three types of feedback in a time estimation task, and their results showed a greater involvement of the ACC and bilateral anterior insula for unexpected positive and unexpected negative feedback, supporting the PRO theory. A growing body of literature has also challenged the RPE hypothesis by pointing out that the striatum, ACC, anterior insula and occipital lobe are activated by both unexpected rewarding and non-rewarding stimuli^20^,^21^,^22^. Jensen *et al.*^23^ directly examined separate brain regions coding for salience and valence. They found a significant SPE signal in the ventral striatum, which was correlated positively with the prediction errors regardless of the stimulus valence. Together, these findings imply that the regions underlying the feedback should be sensitive to the unexpectedness of the feedback and signal a salience prediction error, rather than a reward prediction error.

Due to the relatively low spatial resolution of event-related potentials, the neural sources of positive and negative feedback have not been clearly detected, continuing the controversy between the RPE and SPE hypotheses. Although the PRO model, based on neuroimaging evidence, claims that the mediofrontal cortex (mPFC) is sensitive to the violation of expectancy when learning the action-outcome associations^15^,^16^,^17^, the neural mechanism remains unclear when the actions are irrelevant to the outcomes. In addition, other functional magnetic resonance imaging (fMRI) studies are usually conducted in contexts of classical conditioning or instrumental conditioning^24^,^25^,^26^, investigating the associative learning driven by the PE^27^,^28^, and such studies indeed reflect the processing of associative learning rather than the feedback. Meanwhile, because positive and negative feedback have opposite hedonic valence but are both salient events for survival, it is difficult to differentiate salience from valence in studies investigating neural activities using only appetitive or only aversive events^23^,^24^,^25^,^29^,^30^. Additionally, we noticed that previous studies usually manipulated the prediction by changing the probability of the outcome on a trial-by-trial basis or by varying the outcome’s frequency across blocks of trials, with the assumption that the outcome’s delivery was unexpected in the low probability or infrequent condition^31^,^32^, rather than testing the prediction directly. Therefore, further study is needed to elucidate the neural mechanism of feedback.

In the present study, we adopted the fMRI approach to assess the neural activities of different types of feedback with appetitive and aversive stimuli in one design. To obtain the participants’ accurate predictions, we explicitly informed them of the probabilities that a reward or pain would be delivered in the cue phase and instructed them to press the corresponding buttons to indicate their predictions. This allowed us to accurately divide the unexpected outcomes from the expected outcomes and examine the neural sources of different feedback. Because participants’ precise predictions would vary from trial to trial^31^, we calculated the PE values directly according to their predictions and actual feedback (unlike previous studies that computed the PE indirectly using formulas with additional prior assumptions^22^,^30^,^33^,^34^). Thus, our experimental design clearly investigates the neural mechanisms underlying the feedback and PE. Given the recent findings, we hypothesize that some brain regions related to feedback will treat monetary reward and pain shock similarly as salient events, producing an SPE signal.

## Results

### Behavioral Results

**Figure 1:**
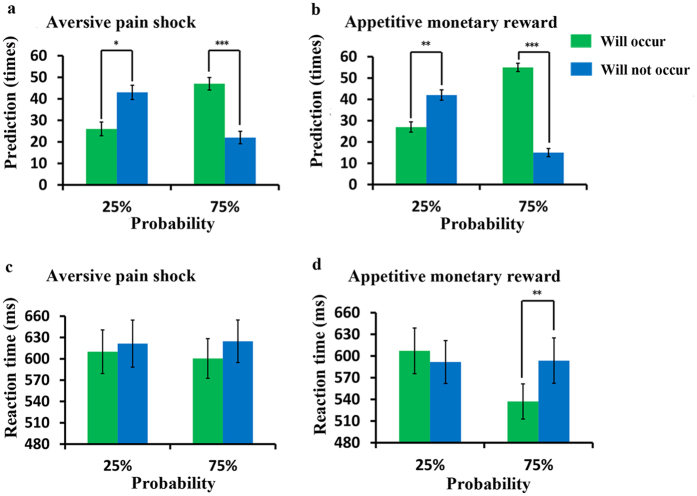
Behavioral results. (**a**) Behavioral responses at the time of the cue for aversive pain shock. Significant differences were found between the two predictions (will occur or will not occur) in both the 25% and 75% probability conditions. (**b**) Behavioral responses at the time of the cue for the appetitive monetary reward. The result was similar to the aversive condition. (**c**) Reaction times for the aversive pain shock. No significant result was found. (**d**) Reaction times for the appetitive monetary reward. As shown, no difference was found in the 25% probability condition between the two predictions, whereas in the 75% probability condition, the reaction time was significantly shorter for “will occur” predictions than “will not occur” predictions. Error bars represent the standard errors. *P < 0.05, **P < 0.01, ***P < 0.001.

First, we performed a repeated-measures ANOVA on behavioral responses at the cue phase with three factors: stimulus (money/pain), probability (25%/75%) and prediction (will or will not occur). The results revealed a significant main effect of prediction (*F*_0.05_ = 35.032, *df* = 1, *P* < 0.0001), but the main effects of stimulus (*P* = 0.061) and probability (*P* = 0.184) were not significant. A significant interaction effect between stimulus and prediction (*F*_0.05_ = 14.241, *df* = 1, *P* = 0.001) and a significant interaction effect between probability and prediction (*F*_0.05_ = 31.985, *df* = 1, *P* < 0.0001) were also revealed, while the interaction effect between stimulus and probability (*P* = 0.26) and the interaction effect among these three factors (*P* = 0.214) were not significant. The subsequent post hoc test showed that for aversive pain, in the 25% probability condition (will occur: mean = 25.80, SEM = 3.19; will not occur: mean = 42.73, SEM = 3.31), the participants tended to press the button predicting that the stimulus would not occur (*P* = 0.014), whereas they exhibited the opposite response (will occur: mean = 46.97, SEM = 2.90; will not occur: mean = 21.67, SEM = 2.89), predicting that the stimulus would occur in the 75% probability condition (*P* < 0.0001; see Fig. 1a). For appetitive money, similarly, the participants mostly predicted that the stimulus would not occur for 25% probability (will occur: mean = 26.73, SEM = 2.42; will not occur: mean = 42.17, SEM = 2.43; *P* = 0.003) and would occur for 75% probability (will occur: mean = 54.73, SEM = 1.96; will not occur: mean = 14.73, SEM = 1.92; *P* < 0.0001; see Fig. 1b).

Next, the reaction time was analyzed using repeated-measures ANOVA. Significant main effects of probability (*F*_0.05_ = 6.137, *df* = 1, *P* = 0.019) and prediction (*F*_0.05_ = 4.698, *df* = 1, *P* = 0.039) were observed, but the main effect of stimulus (*P* = 0.066) was not significant. We then found a significant interaction effect between stimulus and probability (*F*_0.05_ = 10.863, *df* = 1, *P* = 0.003), as well as a significant interaction effect between probability and prediction (*F*_0.05_ = 5.599, *df* = 1, *P* = 0.025), while the interaction effect between stimulus and prediction (*P* = 0.863) and the interaction effect among these three factors (*P* = 0.059) were not significant. In the aversive pain condition, the post hoc test showed no significant difference between “will occur” (mean = 609.97 ms, SEM = 30.65) and “will not occur” (mean = 621.36 ms, SEM = 33.09) predictions in the 25% probability condition (*P* = 0.546); similarly, no difference was found in the 75% probability condition (will occur: mean = 600.45 ms, SEM = 27.95; will not occur: mean = 624.62 ms, SEM = 29.95; *P* = 0.149; see Fig. 1c). In the appetitive money condition, the post hoc test showed no differences between “will occur” (mean = 607.21 ms, SEM = 31.64) and “will not occur” (mean = 591.70 ms, SEM = 29.67) predictions in the 25% probability condition (*P* = 0.264). However, in the 75% probability, the reaction time was significantly shorter (*P* = 0.004) for “will occur” predictions (mean = 537.06 ms, SEM = 24.33) than for “will not occur” predictions (mean = 593.65 ms, SEM = 31.43; see Fig. 1d).

Taken together, we found that for both appetitive and aversive trials, the participants tended to predict that nothing would be presented in the low probability (25%) condition, while the opposite response was observed in the high probability (75%) condition. In addition, after the experiment, most participants reported that their predictions of the upcoming outcomes were mainly based on the probabilities indicated by the cues.

### fMRI Results

The fMRI data were analyzed using two methods: an activation analysis and an analysis of the BOLD responses modulated by the PE. All results are false discovery rate (FDR) corrected, *P* < 0.05.

### **A**ctivation AnaNlyses

First, we performed separate investigations of the regions that responded to different feedback (expected presence, expected absence, unexpected presence, unexpected absence) at the feedback phase. The results are listed separately for the appetitive monetary reward (see Supplementary Table S1) and the aversive pain shock (see Supplementary Table S2). The bilateral putamen (*x*, *y*, *z* = −27, −15, 3, *T* = 4.52; 30, −3, 6, *T* = 4.62), bilateral fusiform gyrus (*x*, *y*, *z* = 33, −48, −15, *T* = 11.93; −36, −48, −21, *T* = 10.29), and bilateral insula (*x*, *y*, *z* = −42, −15, 18, *T* = 7.71; 36, −12, 18, *T* = 6.52) were responsive to expectedly present money; the bilateral middle occipital gyrus (*x*, *y*, *z* = 27, −93, 18, *T* = 8.12; −24, −90, 15, *T* = 5.96) and bilateral middle frontal gyrus (*x*, *y*, *z* = −42, 21, 45, *T* = 5.53; 36, 51, −6, *T* = 4.66) were responsive to expectedly absent money. In addition, we observed activation in the bilateral fusiform gyrus (*x*, *y*, *z* = 36, −51, −15, *T* = 10.15; −36, −45, −21, *T* = 7.8) and bilateral insula (*x*, *y*, *z* = −39, −18, 15, *T* = 4.51; 42, −27, 18, *T* = 4.02) for unexpectedly present money. The right middle occipital gyrus (*x*, *y*, *z* = 24, −90, 15, *T* = 8.46) and bilateral insula (*x*, *y*, *z* = −39, −18, 18, *T* = 6.69; 39, −27, 18, *T* = 4.68) were responsive to unexpectedly absent money. For aversive pain, the bilateral insula (*x*, *y*, *z* = 39, −12, 6, *T* = 8.16; −39, −12, 3, *T* = 6.25) and bilateral postcentral gyrus (*x*, *y*, *z* = 57, −18, 18, *T* = 5.36; −57, −15, 18, *T* = 3.94) were activated when it was expectedly presented; furthermore, the bilateral insula (*x*, *y*, *z* = 45, −15, 3, *T* = 5.84; −39, −9, 6, *T* = 3.62) and bilateral putamen (*x*, *y*, *z* = 30, −3, 3, *T* = 3.93; −27, −3, −3, *T* = 3.21) were activated when it was expectedly absented. We also observed activation in the bilateral insula (*x*, *y*, *z* = 42, −12, 12, *T* = 6.83; −39, −3, 0, *T* = 5.35) and right fusiform gyrus (*x*, *y*, *z* = 36, −48, −15, *T* = 5.42) for unexpectedly present pain and in the bilateral insula (*x*, *y*, *z* = 45, −12, 15, *T* = 6.08; −45, −15, 18, *T* = 4.78) and right cingulate cortex (*x*, *y*, *z* = 18, −42, −3, *T* = 7.45) for unexpectedly absent pain.

**Figure 2:**
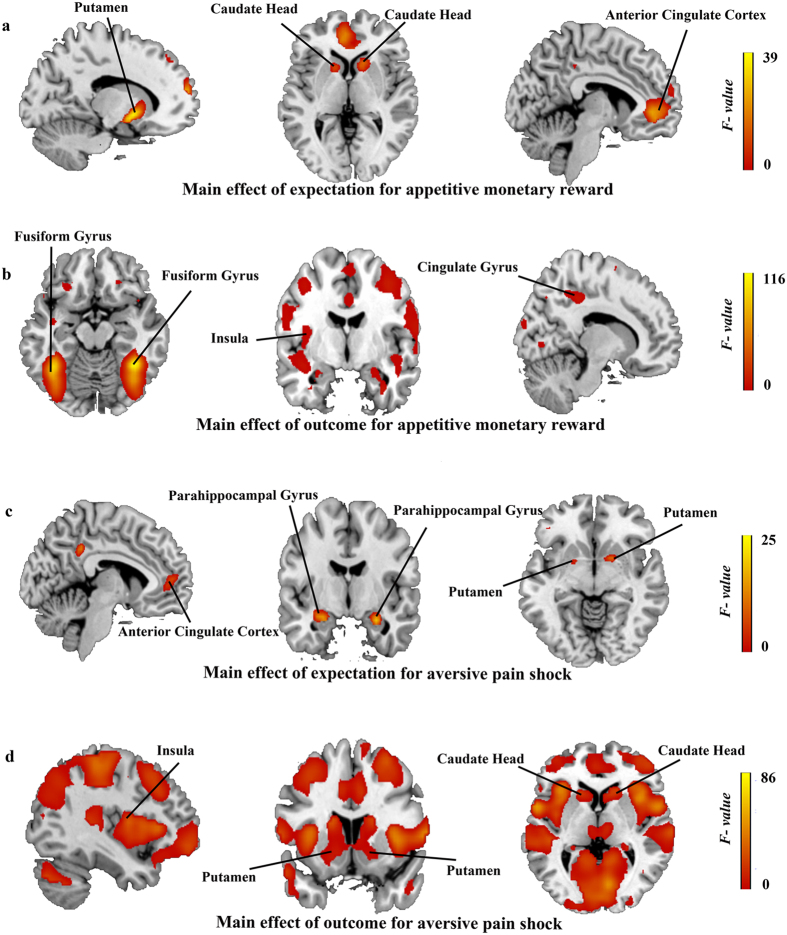
The influence of expectation (expected/unexpected) and outcome (presence/absence) on the feedback. (**a**) The regions that showed higher responses to the expected outcomes than unexpected outcomes for monetary reward. (**b**) The regions that showed higher responses to the present outcomes than absent outcomes for monetary reward. (**c**) The regions showing higher responses to the expected outcomes than unexpected outcomes for pain shock. (**d**) The regions showing higher responses to the present outcomes than absent outcomes for pain shock. The color bar refers to the *F* values. The threshold of statistical significance is P < 0.05, FDR corrected.

ANOVA was then implemented to investigate the influence of expectation (expected/unexpected) and outcome (presence/absence) on the feedback (see Fig. 2). For monetary reward, a significant main effect of the expectation was observed in the bilateral putamen (*x*, *y*, *z* = −15, 6, −6, *F* = 38.37; 15, 12, −3, *F* = 35.71), bilateral caudate head (*x*, *y*, *z* = 12, 18, 3, *F* = 23.19; −9, 15, 3, *F* = 16.4), and bilateral anterior cingulate cortex (*x*, *y*, *z* = −6, 42, 0, *F* = 30.51; 6, 33, 3, *F* = 21.86). The post hoc test revealed higher activities of these regions for expected outcomes than unexpected outcomes. Additionally, a significant main effect of the outcome was found in the bilateral fusiform gyrus (*x*, *y*, *z* = 39, −48, −15, *F* = 115.62; −39, −51, −15, *F* = 102.91), left insula (*x*, *y*, *z* = −36, −6, 15, *F* = 10.26), and right cingulate gyrus (*x*, *y*, *z* = 12, −36, 42, *F* = 18.08). As shown by the post hoc test, the activities of these regions were higher for present outcomes than absent outcomes. For pain shock, the main effect of the expectation was observed in the left anterior cingulate cortex (*x*, *y*, *z* = −6, 42, 3, *F* = 14.13), bilateral parahippocampal gyrus (*x*, *y*, *z* = 27, −9, −18, *F* = 25.01; −24, −12, −18, *F* = 22.91), and bilateral putamen (*x*, *y*, *z* = 18, 6, −6, *F* = 18.49; −18, 3, −6, *F* = 16.08). Similarly, the post hoc test showed that the activities of these regions were higher for expected outcomes than unexpected outcomes. Moreover, the main effect of the outcome was found in the bilateral insula (*x*, *y*, *z* = −39, −3, 9, *F* = 35.61; 39, −3, −3, *F* = 18.57), bilateral putamen (*x*, *y*, *z* = −18, 12, −3, *F* = 9.52; 18, 12, −3, *F* = 7.83), and bilateral caudate head (*x*, *y*, *z* = 15, 21, 3, *F* = 27.21; −15, 18, 3, *F* = 23.94). The post hoc test revealed that the activities of these regions were higher for present outcomes than absent outcomes. No interaction was found for either stimulus (see Table 1 for details).

Under the assumption that the regions related to feedback should treat appetitive and aversive stimuli similarly, as salient events, we conjoined the unexpectedly present money and pain, which revealed an activation of the right fusiform gyrus (*x*, *y*, *z* = 33, −48, −18, *T* = 5.37). In addition, conjoining the unexpectedly absent money and pain revealed the activations of the bilateral insula (*x*, *y*, *z* = 39, −15, 15, *T* = 4.83; −42, −18, 18, *T* = 4.26), right fusiform gyrus (*x*, *y*, *z* = 30, −48, −15, *T* = 3.07) and right posterior cingulate cortex (*x*, *y*, *z* = 12, −60, 3, *T* = 5.69; see Supplementary Table S3).

### Prediction-Error-Related Analyses

This analysis was conducted to investigate the neural activities modulated by the PE when feedback was delivered. We hypothesized that the brain regions consistent with the SPE hypothesis would show a positive correlation between the BOLD signals and PE values for both monetary reward and pain shock.

**Figure 3:**
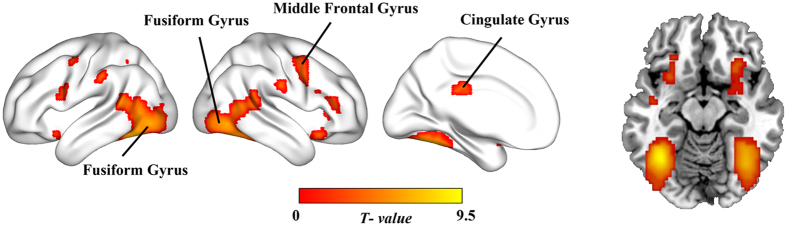
The regions that were modulated positively by the PE for both appetitive and aversive stimuli. Significant activities in bilateral fusiform gyrus, right middle frontal gyrus and left cingulate gyrus were revealed to treat both stimuli similarly as salient events, which was consistent with the SPE hypothesis. The color bar refers to the *T* values. The threshold of statistical significance is P < 0.05, FDR corrected.

The conjunction analysis conjoining the positively modulated activities of the appetitive trials and aversive trials was performed to identify the regions that encoded an SPE signal (see Fig. 3). Large activities were observed in the bilateral fusiform gyrus (*x*, *y*, *z* = −36, −57, −12, *T* = 9.52; 39, −63, −12, *T* = 6.65), right middle frontal gyrus (*x*, *y*, *z* = 45, 6, 57, *T* = 4.34), and left cingulate gyrus (*x*, *y*, *z* = −3, −27, 27, *T* = 3.61). The details are listed in Table 2.

For completeness, another conjunction analysis was conducted to test the RPE hypothesis by conjoining the positively modulated activities of appetitive trials with the negatively modulated activities of aversive trials, but no significant result was found.

## Discussion

In the current study, we investigated the neural activities underlying the feedback with appetitive money and aversive pain shock in one design. As expected, our behavioral results demonstrated that for both money and pain, the participants’ predictions were actually directed by the explicit cue-outcome probabilities indicated by the cue. The fMRI analyses then revealed several brain regions showing higher responses to the expected outcomes than to unexpected outcomes, as well as higher responses to the present outcomes than to absent outcomes, regardless of the stimulus valence. Furthermore, the regions modulated positively by the PE for both stimuli at the feedback phase include the bilateral fusiform gyrus, right middle frontal gyrus, and left cingulate gyrus. In contrast, no significant activity was found to support the RPE hypothesis. These results demonstrate that when feedback is delivered, some regions may treat the appetitive and aversive stimuli similarly as salient events, which is consistent with the SPE hypothesis.

By manipulating the participants’ predictions and real outcomes, four kinds of feedback (expected presence, expected absence, unexpected presence, unexpected absence) were created, making it feasible to accurately investigate the neural mechanism of different feedback types. For both money and pain, there were significantly higher responses in the putamen and anterior cingulate cortex for expected outcomes than unexpected outcomes. It is proposed that the prediction has an important impact on feedback processing, but its role remains unclear. An ERP study found that the FRN was more negative for unexpectedly bad events and that there was little change for expected events^5^; in contrast, Hajcak *et al.*^32^ observed that the FRN was equally large for expected and unexpected negative feedback. Our design made it possible to clearly examine the influence of prediction with a direct measurement, and it revealed higher activities in the putamen and anterior cingulate cortex for expected outcomes than for unexpected outcomes in both appetitive and aversive conditions. The putamen and anterior cingulate cortex have been reliably linked to reward processing^24^,^35^,^36^,^37^, and increased activities have also been found in these regions for aversive stimuli^38^,^39^,^40^. However, because the participants’ predictions have never before been measured directly, it is difficult to compare our results with previous reports.

Moreover, the activity in the insula was higher for present outcomes than absent outcomes, regardless of the stimulus valence. In previous studies, the insula has been reported to be sensitive to various aversive events^30^,^41^, while a study conducted in a context of pain increase and pain relief observed active responses in the insula for both conditions^42^, demonstrating salience encoding in this area. In addition, the certain reward is deemed to be more salient than the certain non-reward^43^, which implies that the feedback signaling the delivery of the outcome may be more salient than the feedback signaling the omission of the outcome.

Importantly, the conjunction analysis showed an SPE signal in the bilateral fusiform gyrus. The fusiform gyrus is a region of the occipital lobe that plays a specific role in processing visual stimuli^44^,^45^. In the current study, substantial PE-related activity was observed in this region when both money and pain were presented unexpectedly. This finding is consistent with a recent fMRI study^22^ in which two types of rewards (pleasant juice and monetary gain) and two types of punishments (aversive juice and aversive picture) were used simultaneously to pinpoint the brain structures responding to the SPE and RPE signals. The results of that study showed that the activation of the bilateral occipital lobe encoded all stimuli similarly, regardless of their valence. Our results further confirmed the SPE signal in this region. Nevertheless, unlike previous studies^22^,^34^, we did not find any SPE signal in the striatum, anterior insula, or ACC. We think that this is not contradictory because our stimuli are quite different from theirs. It has been stated that the SPE signal depends largely upon the type of reinforcement^22^, and the activation of striatum, anterior insula, and ACC was observed only in gustatory SPE analysis (apple juice and salty water), whereas the global SPE analysis, primary SPE analysis and visual SPE analysis did not find any activation of these regions.

Furthermore, our results support a role for the cingulate gyrus in encoding an SPE signal of the feedback. The cingulate gyrus has been identified as a critical area in processing rewards^35^,^46^ and punishments^39^,^47^. A study conducted in the context of pain increase and pain relief also observed an SPE signal in the insula and cingulate gyrus^42^. In addition, the activity of the right middle frontal gyrus was significant when correlated positively with the PE for both stimuli. The middle frontal gyrus has been linked to the PE in appetitive conditioning described by temporal difference learning model^48^. The current study even found a PE-related activity for aversive stimulus, suggesting salience encoding in this area.

All these results uniformly clarify the encoding of salience in the brain regions related to the feedback, which is consistent with the SPE hypothesis. Nevertheless, we cannot ignore the fact that some regions are more sensitive to the appetitive events while others are more sensitive to the aversive events. Instead, we suggest that the processing of the feedback may occur in two stages. The appetitive and aversive events have opposite valence but both are salient events, and the feedback signalling the delivery of the outcome is more salient. Moreover, the feedback containing positive and negative information is presented visually in the current study. Therefore, after the onset of the feedback, the perceptual saliency may be processed rapidly at this early stage when the feedback signals deliver the outcome, but the valence judgement involves a more complex procedure and requires higher cognitive function, which may be processed at a later stage. In addition, the previous studies proposing the RPE signal usually delivered the stimuli to the participants directly^22^,^24^,^25^. Consequently, the intensity of the stimuli would elicit a quick valence judgement, reflecting the processing of the stimuli per se rather than the feedback. Further study including a more accurate design is necessary to investigate the changes of the feedback from the early stage to the late stage.

A limitation of our study is that the nature of the stimuli is different, as the pain shock is primary reinforcement, whereas the monetary reward is secondary reinforcement. Thus, we conducted the analysis independently for the two conditions and do not make any claims about the differences between them. Moreover, it has been demonstrated that the network reflecting the SPE depends largely upon the type of reinforcement^22^. Hence, future studies could further investigate the SPE signal by varying the type of reinforcement (e.g., gustatory and olfactory stimuli). Another limitation is that the actual stimuli were delivered to the participants later than the feedback indicating the outcomes, which may reflect the anticipation of the stimuli at the feedback phase. However, a previous ERP study with a similar design revealed more negative FRN when the feedback cue signalled the unexpected omission of the outcome in both appetitive and aversive conditions^12^, suggesting that the neural activities at the feedback phase may indeed be related to the feedback processing. Further studies are needed to thoroughly investigate the feedback, using a more appropriate design and combining the ERP and fMRI approaches.

In conclusion, the current study advances our understanding of feedback by obtaining the participants’ accurate predictions to elucidate the neural sources of different types of feedback. Significant SPE signal was observed in several regions, suggesting that some regions related to the feedback may treat the appetitive and aversive stimuli similarly as salient events. Feedback plays an important role in decisions and risk-seeking behaviors, as well as in various kinds of addictions^49^,^50^. It has also been proposed that anomalies in the feedback loop may be relevant to schizophrenia^51^. Thus, research on the feedback is genuinely relevant to human health.

## Materials and Methods

### Participants

Thirty-nine participants aged 19–27 years (22 males, mean = 21.6 years, SD = 1.6) were recruited as volunteers. One participant did not complete the procedure because he became uncomfortable inside the scanner. Five more participants were discarded due to large head movements (≥2.5 mm), and three more were discarded because technical problems led to missing behavioral data. The data from the remaining 30 participants (15 males, mean = 21.8 years, SD = 1.6) were subjected to further analyses. All participants were right-handed, with no history of neurological or psychiatric illness, and reported normal or corrected-to-normal vision. The experiment was approved by the Southwest University Human Ethics Committee and was performed in accordance with the guidelines of the Declaration of Helsinki. Written consent was obtained upon each participant’s arrival.

### Stimuli and Apparatus

We investigated neural activities of the feedback with monetary reward and pain shock in one design. The entire experiment was displayed with gray background, and all visual stimuli were presented to the participants through an adjustable mirror above their head when lying on the scanner bed. All visual and electrical stimuli were controlled by E-Prime software (Psychology Tools), which also recorded the behavioral responses and reaction times.

In the monetary reward condition, rewards were displayed as a 10 RMB note, and the participants were told that they would earn a percentage of the cumulative amount in the end. However, the exact percentage was not provided to prevent counting during the experiment^22^. After the experiment, for ethical reasons, all participants earned equal payments of 70 RMB (of which 50 RMB was paid for being scanned and the extra 20 RMB for the reward), but they did not know this amount until the end of the experiment.

In the pain shock condition, a 500 μs aversive painful shock was triggered by a constant current stimulator (DIGITIMER, model DS7A) and administered to the back of the participant’s left hand using an MRI-compatible standard electrode. Upon the participant’s arrival, the pain intensity was titrated individually by increasing the current level incrementally to identify a “painful but tolerable” level, while providing subjective, iterative ratings on a 10-point scale.

### Experimental Procedure

There were two types of stimuli, forming a 2 (type: appetitive money/aversive pain) × 2 (probability: 25%/75%) × 2 (outcome: presence/absence) within-subject design.

**Figure 4:**
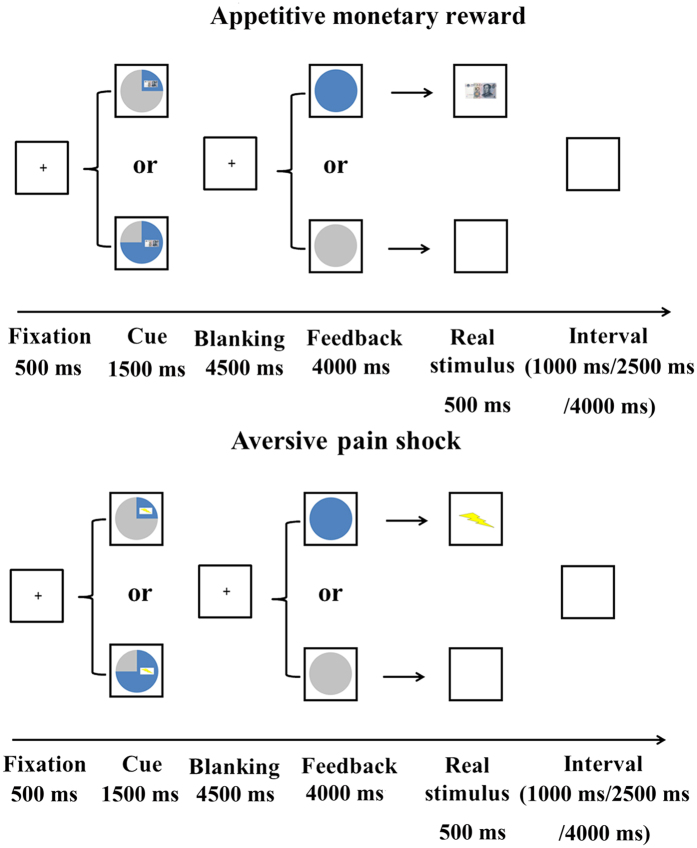
Experimental procedure for the appetitive monetary reward (top) and aversive pain shock (bottom). In both conditions, the participants were instructed to make a prediction by pressing a button at the cue phase. The time course of a trial is designated at the bottom for each condition. See text for details.

Each trial was begun with a cross fixation of 500 ms, after which a pie chart was presented as the cue (1500 ms). The blue-colored portion of the pie chart indicated the probability that the outcome would be delivered, either 25% or 75%, with an image showing either 10 RMB or a lightning bolt, indicating the expected outcome type. Participants were instructed to press the “1” button if they predicted that the stimulus would be presented and to press “2” if they predicted that it would be absent (button assignment was counterbalanced across participants). All participants were informed previously that they should respond as quickly as possible and that the presence or absence of the stimulus was independent of their responses. Then, a cross fixation was displayed for 4500 ms. Feedback (4000 ms) was delivered at the offset of this interval. A completely blue pie chart indicated that the participant would receive the stimulus, whereas a completely gray pie chart indicated that the stimulus would not be delivered. The corresponding stimulus was then presented for 500 ms: an image of 10 RMB in the appetitive condition, an image of a lightning bolt accompanied with cutaneous electrical stimulation in aversive condition, or a gray screen for no stimulus. Finally, a gray screen was presented during the variable-duration intertrial interval (1000 ms, 2500 ms, and 4000 ms; see Fig. 4). After scanning, the participants were asked whether the presence of the stimulus conformed to the probability signalled by the cue, and asked to explain qualitatively how they had made predictions about outcomes.

There were 4 scanning runs each for appetitive and aversive conditions. Each run included 35 trials, resulting in 280 trials in total. Because neural activities involving reward processing are context-dependent^52^, reward and pain trials were divided into separate runs instead of being mixed. The sequence of appetitive and aversive condition runs was counterbalanced across participants. For both conditions, 50% of the trials were followed by actual stimuli, and 50% were followed by a gray screen. The trial order was pseudorandom. The entire scanning process lasted approximately 1 hour.

### fMRI Image Acquisition and Preprocessing

The fMRI data were acquired in a 3-Tesla Siemens MRI scanner with gradient echo T_2_^*^-weighted echo-planar imaging sequence to measure the blood oxygenation level dependent (BOLD) signal. Using a standard head coil, 25 oblique-axial slices parallel to the AC-PC line were obtained per volume (TR = 1.5 s, TE = 29 ms, FOV = 192 × 192 mm^2^, flip angle = 90°, matrix = 64 × 64, slice thickness = 5 mm). In total, 319 volumes were acquired for each run, at an ascending continuous sequence. Moreover, high-resolution T_1_-weighted structural images (1 × 1 × 1 mm^3^) were acquired at the beginning of the scan.

Data preprocessing was conducted using the software package SPM8. The first 5 volumes of each run were removed to reach a steady magnetic field, after which the functional images were corrected for slice timing and realignment. Subsequently, all images were normalized into the Montreal Neurological Institute (MNI) space using unified segmented T1 images with a resampled voxel size of 3 × 3 × 3 mm^3^. Finally, spatial smoothness was performed using a Gaussian kernel of 8 mm, with full-width at half-maximum.

### fMRI Data Analyses

First, we conducted an activation analysis to obtain the response patterns of different feedback. Then, an approach using the PE values as parametric modulators was implemented to investigate the PE-related activities. Note that the appetitive money and aversive pain were scanned and analyzed separately to avoid context-dependent effects.

### Activation Analyses

In the current study, according to the participants’ predictions and actual outcomes, four kinds of feedback were created (i.e., expected presence, expected absence, unexpected presence, and unexpected absence). For instance, when the participant’s prediction was “will occur” and the stimulus was actually delivered, it was an “expected presence”; when the participant’s prediction was “will occur” and the stimulus was omitted, it was an “unexpected absence”. Similarly, when the participant’s prediction was “will not occur” and the stimulus was actually delivered, it was an “unexpected presence”; when the participant’s prediction was “will not occur” and the stimulus was omitted, it was an “expected absence”.

The whole-brain statistical analysis was performed using the general linear model. At the feedback phase, the time was locked to the onset of feedback, and the four kinds of feedback (expected presence, expected absence, unexpected presence, and unexpected absence) were respectively modeled with a duration of 4 s. In addition, 6 ongoing motion parameters obtained during realignment were included but were of no interest. All of these 10 regressors were convolved with the canonical hemodynamic response function and restricted to a whole-brain grey matter mask.

For first-level analysis, we calculated the single-subject contrasts for each of these four kinds of feedback using the whole-brain activations as the baseline. Then, to identify the activations related to different feedback types, we performed four second-level one-sample t-tests for the four kinds of feedback based on the single-subject contrasts. In addition, a full factorial design that included expectation (expected/unexpected) and outcome (presence/absence) was subsequently conducted to investigate the influence of these two factors.

Furthermore, we also performed a conjunction of unexpectedly present money and unexpectedly present pain, as well as a conjunction of unexpectedly absent money and unexpectedly absent pain, to search for the brain regions responding similarly to appetitive and aversive events.

### Prediction-Error-Related Analyses

The participants were instructed to make a prediction at the cue phase, and the stimuli were delivered in 50% of trials, leading to positive (better than expected) and negative (worse than expected) prediction errors throughout the trials. Our design made it feasible to calculate the PE values directly by obtaining the participants’ predictions and actual values. The predicted value was arbitrarily assigned to 1 when the prediction was “will occur” and to 0 for the “will not occur” prediction. Moreover, the actual value was set as 1 when the stimulus was actually presented and as 0 when it was absent. The calculated PE values were then used as parametric modulators to investigate the PE-related activities. According to the participants’ predictions and actual values, we divided the outcomes into the expected and unexpected outcomes. Note that our subsequent analysis focused mainly on the unexpected outcomes because there was no prediction error in the expected condition. Thus, the unexpected feedback and corresponding PE values were modeled with a duration of 4 s, as the time was locked to the onset of feedback. Including the 6 motion parameters, all 8 regressors were convolved with a canonical hemodynamic response function and restricted to a whole-brain grey matter mask. Our first-level contrasts were calculated as follows: (1) appetitive money’s unexpected presence and absence positively modulated by the PE; (2) aversive pain’s unexpected presence and absence positively modulated by the PE; (3) aversive pain’s unexpected presence and absence negatively modulated by the PE.

The second-level analyses included two conjunction analyses to examine the SPE and RPE hypotheses at the feedback phase. For SPE hypothesis, which suggests that some regions would be modulated by the PE positively for both money and pain, showing similar activities in the two conditions, we conjoined the contrasts 1 and 2. Next, corresponding to the RPE hypothesis that some regions would be modulated by the PE positively for money but negatively for pain, showing opposite activities in the two conditions, a conjunction analysis including contrasts 1 and 3 was performed.

## Additional Information

**How to cite this article**: Gu, Y. *et al.* Neural Activities Underlying the Feedback Express Salience Prediction Errors for Appetitive and Aversive Stimuli. *Sci. Rep.* **6**, 34032; doi: 10.1038/srep34032 (2016).

## Supplementary Material

Supplementary Information

## Figures and Tables

**Table 1:** ANOVA results at the feedback phase for appetitive money and aversive pain shock at *p* < 0.05 (FDR corrected).

Regions	Laterality	Voxels	x	y	z	*F*
Main effect of expectation for appetitive monetary reward						
Putamen	L	96	−15	6	−6	38.37
Putamen	R	90	15	12	−3	35.71
Superior Frontal Gyrus	L	343	−15	57	18	31.25
Superior Frontal Gyrus	R	78	18	42	45	16.52
Anterior Cingulate Cortex	L	190	−6	42	0	30.51
Anterior Cingulate Cortex	R	122	6	33	3	21.86
Caudate Head	R	114	12	18	3	23.19
Caudate Head	L	85	−9	15	3	16.40
Posterior Cingulate Cortex	L	91	−3	−36	42	21.15
Main effect of outcome for appetitive monetary reward						
Fusiform Gyrus	R	348	39	−48	−15	115.62
Fusiform Gyrus	L	301	−39	−51	−15	102.91
Inferior Occipital Gyrus	R	137	39	−69	−9	114.77
Inferior Occipital Gyrus	L	334	−36	−76	−9	100.68
Inferior Frontal Gyrus	R	572	48	6	30	51.81
Middle Occipital Gyrus	L	467	−42	−75	3	22.15
Cingulate Gyrus	R	239	12	−36	42	18.08
Visual Cortex	R	67	12	−93	18	12.59
Insula	L	83	−36	−6	15	10.26
Main effect of expectation for aversive pain shock						
Parahippocampal Gyrus	R	78	27	−9	−18	25.01
Parahippocampal Gyrus	L	63	−24	−12	−18	22.91
Superior Frontal Gyrus	L	174	−18	42	42	19.86
Cingulate Gyrus	L	46	−6	−42	36	19.13
Putamen	R	47	18	6	−6	18.49
Putamen	L	15	−18	3	−6	16.08
Anterior Cingulate Cortex	L	60	−6	42	3	14.13
Main effect of outcome for aversive pain shock						
Precentral Gyrus	L	335	−57	6	9	85.95
Precentral Gyrus	R	358	57	6	9	82.50
Inferior Parietal Lobule	L	440	−63	−27	24	64.39
Inferior Parietal Lobule	R	324	48	−45	54	25.14
Insula	L	303	−39	−3	9	35.61
Insula	R	310	39	−3	−3	18.57
Posterior Cingulate Cortex	R	40	9	−54	6	33.08
Posterior Cingulate Cortex	L	53	−9	−54	6	32.33
Caudate Head	R	165	15	21	3	27.21
Caudate Head	L	156	−15	18	3	23.94
Supplementary Motor Cortex	R	85	12	0	66	25.79
Anterior Cingulate Cortex	R	74	9	12	36	22.75
Thalamus	R	79	6	−18	0	21.66
Midbrain	R	151	6	−30	−6	21.18
Putamen	L	96	−18	12	−3	9.52
Putamen	R	54	18	12	−3	7.83

Abbreviations: L, left hemisphere; R, right hemisphere; FDR, false discovery rate; x, y, z, the peak coordinates in MNI space.

**Table 2:** Regions that were modulated positively by the PE for both monetary reward and pain shock (FDR corrected, *p* < 0.05).

Regions	Laterality	Voxels	x	y	z	*t*
Fusiform Gyrus	L	197	−36	−57	−12	9.52
Fusiform Gyrus	R	184	39	−63	−12	6.65
Middle Occipital Gyrus	L	181	−39	−75	−6	6.11
Middle Temporal Gyrus	R	186	51	−57	6	5.15
Middle Frontal Gyrus	R	89	45	6	57	4.34
Superior Temporal Gyrus	R	66	66	−45	15	4.11
Postcentral Gyrus	R	34	69	−18	27	4.06
Inferior Frontal Gyrus	R	31	45	33	12	3.89
Inferior Frontal Gyrus	L	39	−57	12	21	3.72
Cingulate Gyrus	L	48	−3	−27	27	3.61

Abbreviations: L, left hemisphere; R, right hemisphere; FDR, false discovery rate; x, y, z, the peak coordinates in MNI space.
