# Supplementary material for: Neural Activities Underlying the Feedback Express Salience Prediction Errors for Appetitive and Aversive Stimuli
Source: Sci Rep. 2016 Oct 3;6:34032. doi: 10.1038/srep34032 (PMC5046116; doi:10.1038/srep34032)
Supplement: Supplementary Information [file srep34032-s1.pdf]

Neural Activities Underlying the Feedback Express Salience Prediction Errors for Appetitive and Aversive Stimuli

Yan Gu[1], Xueping Hu[1], Weigang Pan[1], Chun Yang[1], Lijun Wang[1], Yiyuan Li[2] and Antao Chen[1]*

Supplementary Table S1. Regions that were activated at the feedback phase in monetary reward condition at p < 0.05 (FDR corrected). Abbreviations: L, left hemisphere; R, right hemisphere; FDR, false discovery rate; x, y, z, the peak coordinates in MNI space.

Regions	Laterality	Voxels	x	y	z	*t*
**Regions for expectedly present money**						
Fusiform Gyrus	R	434	33	-48	-15	11.93
Fusiform Gyrus	L	434	-36	-48	-21	10.29
Inferior Occipital Gyrus	R	103	36	-72	-9	10.23
Insula	L	98	-42	-15	18	7.71
Insula	R	212	36	-12	18	6.52
Amygdala	R	46	24	-6	-18	6.63
Amygdala	L	40	-24	-3	-18	5.27
Posterior Cingulate Cortex	L	357	12	-57	6	6.04
Anterior Cingulate Cortex	L	181	-9	42	-3	4.85
Anterior Cingulate Cortex	R	73	3	39	0	3.98
Putamen	R	160	30	-3	6	4.62
Putamen	L	104	-27	-15	3	4.52
**Regions for expectedly absent money**						
Middle Occipital Gyrus	R	228	27	-93	18	8.12
Middle Occipital Gyrus	L	151	-24	-90	15	5.96
Visual Cortex	L	410	-24	-96	18	7.34
Fusiform Gyrus	L	248	-21	-51	-12	7.09
Middle Frontal Gyrus	L	513	-42	21	45	5.53
Middle Frontal Gyrus	R	177	36	51	-6	4.66
Superior Frontal Gyrus	L	342	-18	45	33	5.35
Putamen	L	99	-27	-12	6	4.30
Putamen	R	115	30	-9	0	4.28
**Regions for unexpectedly present money**						

Inferior Occipital Gyrus	R	87	36	-72	-9	10.66
Fusiform Gyrus	R	409	36	-51	-15	10.15
Fusiform Gyrus	L	325	-36	-45	-21	7.80
Middle Occipital Gyrus	R	318	27	-90	15	7.32
Superior Parietal Lobule	R	201	30	-60	60	6.95
Posterior Cingulate Cortex	R	241	18	-63	9	5.35
Parahippocampal Gyrus	R	402	30	-24	-18	4.93
Insula	L	92	-39	-18	15	4.51
Insula	R	139	42	-27	18	4.02
**Regions for unexpectedly absent money**						
Middle Occipital Gyrus	R	158	24	-90	15	8.46
Visual Cortex	L	346	-12	-102	9	7.73
Insula	L	107	-39	-18	18	6.69
Insula	R	139	39	-27	18	4.68
Parahippocampal Gyrus	R	312	12	-42	-3	5.35
Posterior Cingulate Cortex	L	246	6	-75	21	4.91
Inferior Parietal Lobule	R	224	51	-42	51	3.93

Supplementary Table S2. Regions that were activated at the feedback phase in pain shock condition (FDR corrected, $p<0.05$). Abbreviations: L, left hemisphere; R, right hemisphere; FDR, false discovery rate; x, y, z, the peak coordinates in MNI space.

Regions	Laterality	Voxels	x	y	z	*t*
**Regions for expectedly present pain shock**						
Precentral Gyrus	R	116	57	0	6	8.69
Insula	R	279	39	-12	6	8.16
Insula	L	223	-39	-12	3	6.25
Fusiform Gyrus	R	107	36	-48	-15	5.72
Postcentral Gyrus	R	162	57	-18	18	5.36
Postcentral Gyrus	L	115	-57	-15	18	3.94
**Regions for expectedly absent pain shock**						
Visual Cortex	L	319	-12	-102	9	7.82
Middle Occipital Gyrus	R	193	18	-96	9	6.67
Insula	R	144	45	-15	3	5.84
Insula	L	105	-39	-9	6	3.62
Parahippocampal Gyrus	L	518	30	-21	-18	4.60
Posterior Cingulate Cortex	R	229	6	-66	12	4.22
Middle Frontal Gyrus	R	85	30	30	51	4.21
Middle Frontal Gyrus	L	100	-36	24	51	3.98
Putamen	R	94	30	-3	3	3.93
Putamen	L	87	-27	-3	-3	3.21
**Regions for unexpectedly present pain shock**						
Precentral Gyrus	R	109	57	0	9	7.61
Precentral Gyrus	R	75	-48	-9	12	5.12
Insula	R	219	42	-12	12	6.83
Insula	L	163	-39	-3	0	5.35
Fusiform Gyrus	R	74	36	-48	-15	5.42

**Regions for unexpectedly absent pain shock**						
Visual Cortex	L	279	-12	-102	9	7.52
Cingulate Cortex	R	122	18	-42	-3	7.45
Middle Occipital Gyrus	R	127	18	-99	12	6.22
Insula	R	96	45	-12	15	6.08
Insula	L	125	-45	-15	18	4.78
Posterior Cingulate Cortex	R	221	12	-66	12	5.06
Posterior Cingulate Cortex	L	80	-6	-72	18	4.64
Middle Temporal Gyrus	L	110	-57	-33	0	4.11

Supplementary Table S3. Regions that were activated similarly in both monetary reward and pain shock conditions (FDR corrected, p<0.05). Abbreviations: L, left hemisphere; R, right hemisphere; FDR, false discovery rate; x, y, z, the peak coordinates in MNI space.

Regions	Laterality	Voxels	x	y	z	*t*
**Conjunction of unexpectedly present money and unexpectedly present pain**						
Fusiform Gyrus	R	25	33	-48	-18	5.37
**Conjunction of unexpectedly absent money and unexpectedly absent pain**						
Visual Cortex	L	290	-12	-102	9	8.02
Middle Occipital Gyrus	R	47	24	-90	15	7.65
Posterior Cingulate Cortex	R	219	12	-60	3	5.69
Cingulate Gyrus	R	118	15	-42	-3	5.60
Insula	R	134	39	-15	15	4.83
Insula	L	102	-42	-18	18	4.26
Parahippocampal Gyrus	R	52	21	-51	-6	4.49
Inferior Parietal Lobule	R	68	51	-48	54	4.04
Superior Temporal Gyrus	L	25	-66	-27	6	3.81
Superior Temporal Gyrus	R	39	66	-18	0	3.23
Fusiform Gyrus	R	178	30	-48	-15	3.07
